# Heterogeneity in NK Cell Subpopulations May Be Involved in Kidney Cancer Metastasis

**DOI:** 10.1155/2022/6378567

**Published:** 2022-08-22

**Authors:** Zhengfang Liang, Fengwei Nong, Jingjie Zhao, Dalong Wei, Qianli Tang, Jian Song, Lingzhang Meng

**Affiliations:** ^1^Jinan University, Guangzou, Guangdong Province, China; ^2^Center for Systemic Inflammation Research (CSIR), School of Preclinical Medicine, Youjiang Medical University for Nationalities, Baise, Guangxi Province, China; ^3^Department of Urinary Surgery, The Affiliated Hospital of Youjiang Medical University for Nationalities, Baise, Guangxi Province, China; ^4^Department of Renal Diseases, The Affiliated Hospital of Youjiang Medical University for Nationalities, Baise, Guangxi Province, China; ^5^Life Science and Clinical Research Center, The Affiliated Hospital of Youjiang Medical University for Nationalities, Baise, China; ^6^Burn Plastic & Trauma Surgery Department, The Affiliated Hospital of Youjiang Medical University for Nationalities, Baise, Guangxi Province, China; ^7^Institute of Cardiovascular Sciences, Guangxi Academy of Medical Sciences, Nanning, Guangxi Province, China

## Abstract

Although substantial progress has been made in the immunotherapy of kidney cancer, its efficacy varies from patient to patient, with many responding suboptimally or even developing metastases. Thus, research on the tumour immune microenvironment and immune cell heterogeneity is essential for kidney cancer treatment. In this study, natural killer (NK) cell populations were isolated using signature genes from the single-cell sequencing data of clear cell renal cell carcinoma (ccRCC) and normal kidney tissues and divided into three subpopulations according to the differences in gene expression profiles: NK(GZMH), NK(EGR1), and NK(CAPG). Gene set enrichment analysis revealed that NK(EGR1) and NK(CAPG) were closely related to tumour metastasis, as shown by kidney cancer metastasis to Hodgkin lymphoma, T-cell leukaemia, and Ki-1+ anaplastic large cell lymphoma. Thus, these two NK cell subpopulations are promising targets for inhibiting metastasis in ccRCC. Our findings revealed heterogeneity in the infiltrating NK cells of kidney cancer, which can serve as a reference for the mechanisms underlying metastasis in kidney cancer.

## 1. Introduction

Kidney cancer is one of the most common malignancies, ranking ninth among male malignancies and 14th among female malignancies [1]. More than 400,000 new kidney cancer cases are diagnosed worldwide each year, accounting for 2.2% global burden of all cancers [2]. Clear cell renal cell carcinoma (ccRCC) is the most common type of renal cell carcinoma, accounting for over 80% cases [3]. Presently, surgery is the main treatment for ccRCC, whereas adjuvant interleukin 2 (IL-2) therapy, interferon (IFN) therapy, chemotherapy, radiotherapy, and hormonal therapy for ccRCC at high recurrence risk do not prolong overall patient survival [4]. Many patients are treated with a single approach (predominantly surgery) without adjuvant treatment for tumour recurrence and metastasis, while some patients experience tumour recurrence, progression, and metastasis, which can affect their therapeutic efficacy and survival quality. Therefore, exploring the mechanisms of progression and metastasis in ccRCC is crucial for its treatment.

Recently, tumour immunotherapy has demonstrated strong antitumour activity in several tumours and been shown to be effective in ccRCC treatment [5]. Therefore, understanding the tumour microenvironment and tumour immune cell infiltration can help to elucidate the mechanisms of tumour growth, progression, and metastasis. Tumour infiltration of B cells is associated with poor prognosis and distant metastasis in ccRCC [6]. CCL5, a marker associated with CD8+ T-cell infiltration, promotes the proliferation and invasive capacity of ccRCC cell lines [7]. Moreover, neutrophil phenotype and function can be affected by kidney cancer-related inflammation, leading to metastasis through the high expression of CXC chemokines [8], while neutrophils may also be involved in immunotherapy resistance [9]. Although natural killer (NK) cells are important immune cells in the body that play a vital role in tumour immunity, research on the status of NK cells in the tumour microenvironment and NK cell heterogeneity, in particular, is relatively scarce.

NK cells are cytotoxic lymphocytes that play a crucial role in tumour immune surveillance. Studies have shown that NK cells are closely associated with many types of cancer, and the degree of NK cell infiltration is related to patient survival [10]. Although some kidney cancer patients have benefitted from immunotherapy, not all patients respond to current immunotherapies, and some studies have found a high NK cell infiltration in kidney cancer, suggesting that NK cells are heterogeneous in kidney cancer, which may affect the efficacy of immunotherapy [11]. Exploring the heterogeneity of NK cells in infiltrating ccRCC can help us to understand the mechanisms underlying kidney cancer development and immune escape. In this study, NK cells were identified in ccRCC specimens, and three subtypes were isolated. Further analysis revealed that two of these subtypes promoted colorectal cancer, breast cancer, Hodgkin's lymphoma, T-cell leukaemia, and Ki-1+ anaplastic large cell lymphoma.

## 2. Materials and Methods

### 2.1. scRNA-Seq Bioinformatic Analysis

The scRNA-seq data of ccRCC and normal kidney tissues were retrieved from the NCBI GEO database (https://www.ncbi.nlm.nih.gov/geo/) under the accession codes GSE121636 [12] and GSE131685 [13], respectively. SCTransform wrapper was used to minimise the technical variations between different panels and platforms. R package Seurat (v4.0.2) was used to isolate the cell cluster at a resolution of 0.7, gene nebula maps were used to show the specific gene expression levels, and R package EnhancedVolcano (v1.11.3) was used to show the differentially expressed genes (DEGs). After calculation, the DEGs with ∣Log2FC | >1 and *p* < 0.05 were considered as significant ones. The R package clusterProfiler (v4.0.0) was used to perform gene set enrichment analysis (GSEA).

### 2.2. Human Biopsies

The cancerous biopsies were isolated from ccRCC patients by surgery. After pathological examination, the redundant tissues were used for this study. The written informed consent was provided by the patients. This study was approved by the Ethics Committee of Youjiang Medical University for Nationalities.

### 2.3. Flow Cytometry

The surgically isolated ccRCC cancerous tissues were used for the preparation of single-cell suspensions after digestion with collagenase IV (30 mg/mL, Gibco, #17104-019). To remove debris, the samples were filtered through a 70 *μ*m cell strainer. After counting, 1 × 10^6^ cells were used for staining for each sample, and the cells were suspended with MACS buffer (Miltenyi Biotec, #130-091-221). After blocking the potential unspecific binding sites, the cells were performed surface and intracellular staining with FITC anti-human CD16 (Biolegend, #980112), APC anti-human CD56 (Biolegend, #981204), Alexa Fluor 405 anti-human GZMH (GBiosciences, #ITT2050-405), PE anti-human EGR1 (Invitrogen, # 12-9851-42), and anti-human/anti-mouse CAPG (Creative Biolabs, #CBMAB-C0844-FY). The CAPG antibody was conjugated with Alexa Fluor 700 (Invitrogen, #A20110). The dead cells were counterstained with fixable viability dye. The stained samples were resuspended with PBS/0.5%BSA/2 mM EDTA buffer and recorded on a flow cytometer (ThermalFisher Attune Nxt).

## 3. Results

### 3.1. NK Cell Identification

**Figure 1:**
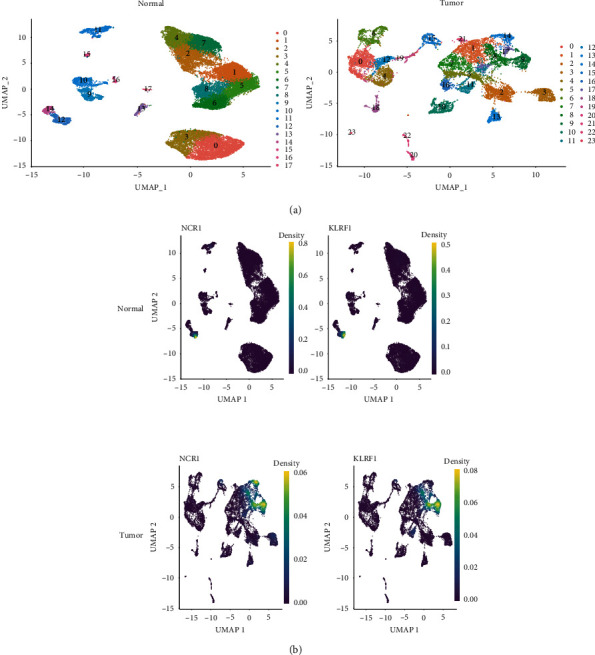
Isolation and identification of natural killer (NK) cells. (a) UMAP plot showing 18 cell types in normal human kidney tissues and 24 cell types in clear cell renal cell carcinoma (ccRCC). (b) Nebulograms showing that NK cell markers (NCR1 and KLRF1) are preferentially expressed in cluster 12 in normal kidney tissues and clusters 8, 9, 14, and 17 in ccRCC.

The genes from normal human kidney cells were grouped into 18 clusters with the retrieved scRNA-seq data, while those from ccRCC immune cells were grouped into 24 clusters (Figure 1(a)). NCR1 and KLRF1 are used to identify NK cells [14, 15], which were preferentially expressed in cluster 12 in normal kidney cells and clusters 8, 9, 14, and 17 in ccRCC (Figure 1(b)), indicating these clusters contain genes from the NK cells.

### 3.2. Human Kidney and ccRCC Cells Are Heterogeneous

**Figure 2:**
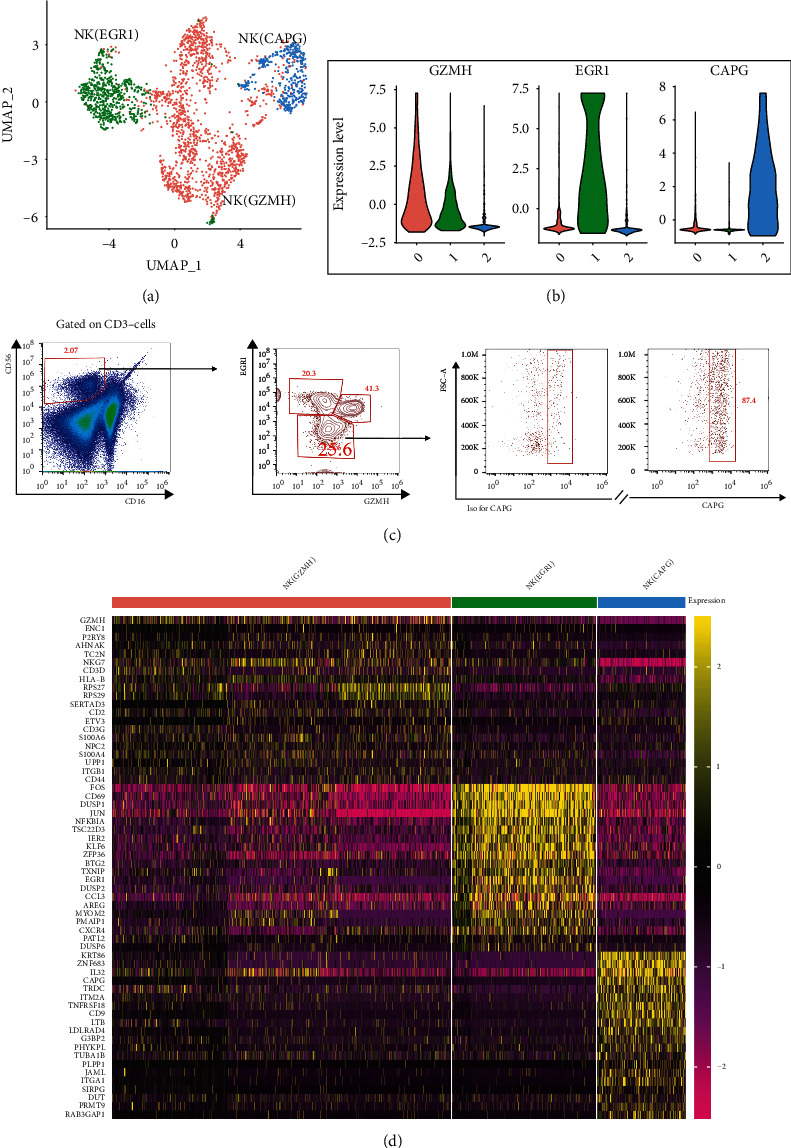
scRNA-seq analysis showing three natural killer (NK) cell subpopulations. (a) UMAP plot showing the clustering in normal kidney cells and clear cell renal cell carcinoma (ccRCC). Three subpopulations were identified based on genetic mapping. (b) Violin plot showing the preferential expressions of the signature genes GZMH, EGR1, and CAPG in three NK cell subpopulations, respectively. (c) Flow cytometric validation of NK subpopulations. NK cells were identified as CD3-CD16-CD56+ cells and were subdivided by counterstaining of antibodies against EGR1, GZMH, and CAPG. (d) Heat map showing the expression patterns of the top 20 genes in each NK cell subpopulation.

To compare the biological characteristics of NK cell subpopulations of kidney cancer and normal kidney tissues, NK cells were isolated from normal kidney and tumour tissues and then divided into subpopulations according to the differences in gene expression profiles. Three subpopulations were obtained (Figure 2(a)), which preferentially expressed GZMH, EGR1, and CAPG, respectively (violin plots, Figure 2(b)). A comparative analysis of the gene expression profiles of the three subpopulations revealed that 212, 192, and 271 genes were preferentially expressed in NK(GZMH), NK(EGR1), and NK(CAPG) subpopulations, respectively. To confirm the classification of NK subpopulations by scRNA-seq, the ccRCC biopsies were isolated from patients and underwent further analysis (Figure 2(c)). The three subpopulations could be validated by flow cytometry (Figure 2(c)). The top 20 gene expressions of each cluster are depicted as heat maps (Figure 2(d)).

### 3.3. NK Cell Subpopulations Promote Kidney Cancer Metastasis

**Figure 3:**
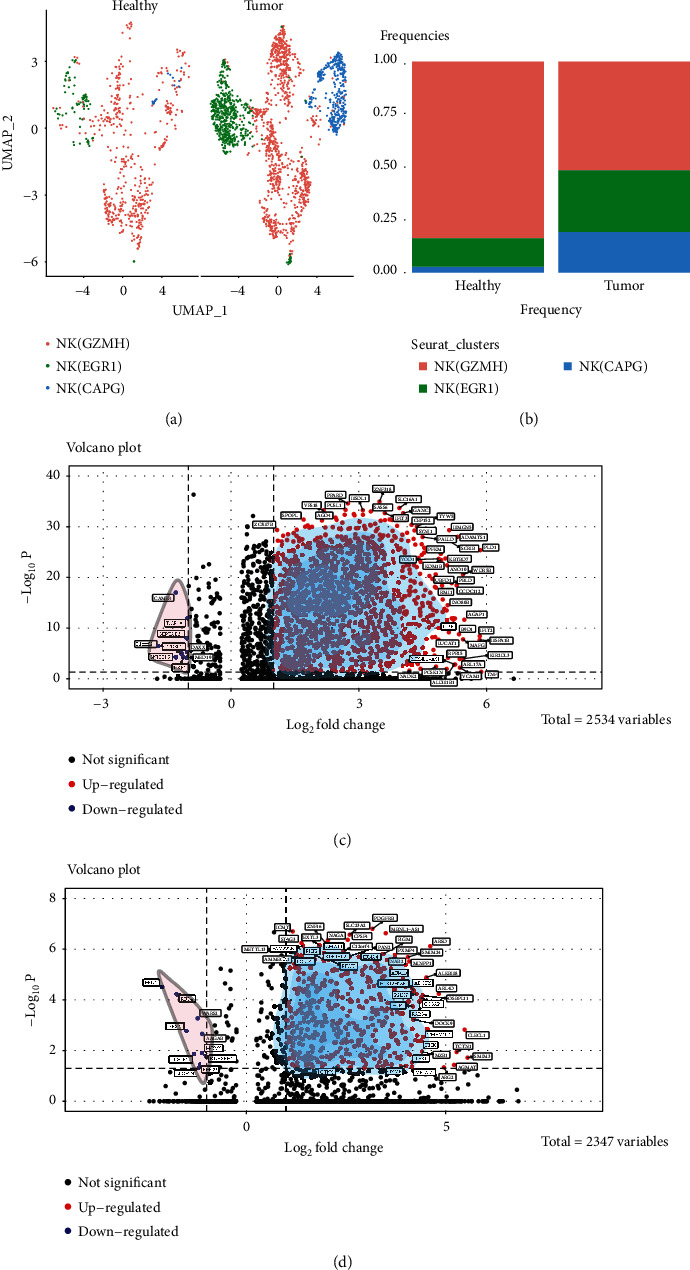
Comparison of natural killer (NK) cell subpopulations between normal kidney and kidney cancer tissues. (a) Split UMAP plots showing the distribution of NK cell subpopulations in normal kidney tissues and clear cell renal cell carcinoma (ccRCC). (b) Stacked bar graph showing the frequency of NK cell subpopulations (normal tissues vs. ccRCC). (c) Volcano plot showing differentially expressed genes (DEGs) in the NK cell subpopulation NK(EGR1) between ccRCC and normal kidney tissues. (d) Volcano plot showing DEGs in the NK cell subpopulation NK(CAPG) between ccRCC and normal kidney tissues.

**Figure 4:**
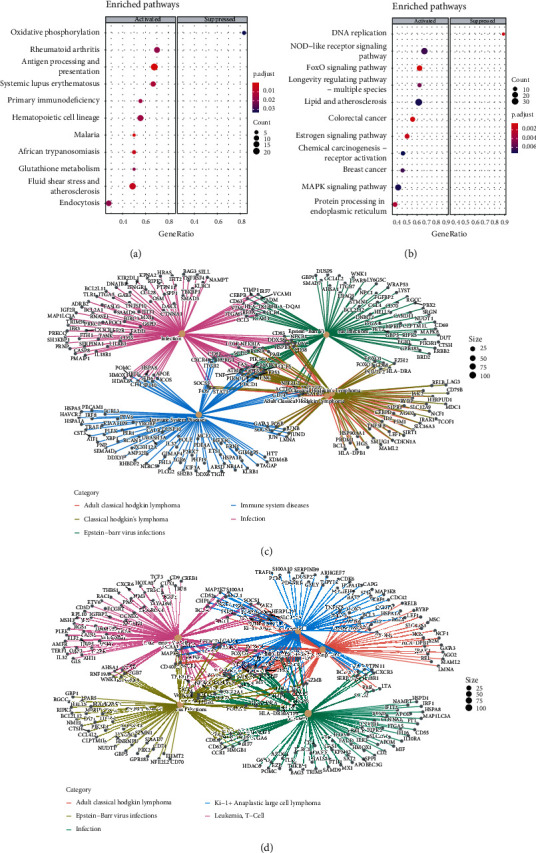
Functional analysis of natural killer (NK) cell subpopulations NK(EGR1) and NK(CAPG). (a) Dot plot showing the up-/downregulated pathways of NK(EGR1). (b) Dot plot showing the up-/downregulated pathways of NK(CAPG). (c)CNET plot showing the depth of NK(EGR1) and metastasis prediction. (d) CNET plot showing the depth of NK(CAPG) and metastasis prediction.

A comparison of subpopulation characteristics between normal kidney tissues and ccRCC revealed that the proportion of cells in the NK(EGR1) and NK(CAPG) subpopulations was significantly higher in ccRCC than that in these subpopulations in normal kidney tissues (Figures 3(a) and 3(b)), thus suggesting that the NK(EGR1) and NK(CAPG) subpopulations play a facilitatory role in tumourigenesis. Therefore, the NK(EGR1) and NK(CAPG) subpopulations were the focus of this study. Further comparative analysis of gene expression differences between these two subgroups in kidney cancer and normal kidney tissues yielded 2,534 DEGs in NK(EGR1), of which 1,306 were significantly upregulated and 9 were significantly downregulated (Figure 3(c)). NK(CAPG) had 2,347 DEGs, of which 430 were significantly upregulated and 10 were significantly downregulated (Figure 3(d)). GSEA revealed that NK(EGR1) inhibited oxidative phosphorylation (Figure 4(a)), while relevant reports have confirmed that oxidative phosphorylation is significantly reduced in many solid tumours [16]. NK(EGR1) is upregulated in primary immunodeficiency (Figure 4(a)), which in turn is associated with tumourigenesis in several cancers [17, 18]. The GSEA findings indicate that NK(CAPG) promotes the FoxO and MAPK signalling pathways (Figure 4(b)), both of which are involved in various carcinogenic mechanisms [19, 20]. Furthermore, NK(CAPG) also promotes colorectal cancer and breast cancer (Figure 4(b)). These results suggest that NK(EGR1) and NK(CAPG) play a role in promoting cancer. Gene correlation network analysis of DEGs in these two NK subpopulations yielded the following results: NK(EGR1) promotes kidney cancer metastasis to Hodgkin's lymphoma (Figure 4(c)), while NK(CAPG) promotes metastasis to T-cell leukaemia, Ki-1+ anaplastic large cell lymphoma, and adult classical Hodgkin's lymphoma (Figure 4(d)).

## 4. Discussion

Metastasis is a common issue that frequently occurs in the tumour treatment, with up to 17% renal cell carcinomas exhibiting distant metastases during diagnosis [21]. Therefore, surgery alone is currently not sufficient to treat kidney cancer. However, kidney cancer is also insensitive to radiotherapy and chemotherapy, which has prompted scientists to continue exploring new treatment strategies. Immunomodulators (IL-2 and IFN-alpha) have limited effectiveness in treating metastatic ccRCC [22]. Targeted drug therapy is effective in treating metastatic ccRCC, particularly in cases of pancreatic metastases, but less effective in metastases to other organs [23]. The in-depth investigations on the tumour immune microenvironment have led to the rapid development of tumour immunotherapy, which is now a research hotspot in tumour treatment. The discovery of the PD-1 and CTLA-4 signalling pathways gave rise to new advances in immunotherapy for ccRCC [24, 25]. However, due to its limitations, a significant number of patients have exhibited poor outcomes [25, 26]. Therefore, there is still a need to explore the tumour immune microenvironment and immune cell infiltration, to combat tumour escape and immune cell-mediated metastasis.

NK cells have long been considered to have tumour suppressive effects (including in ccRCC) [10, 27, 28], but research on their heterogeneity has been scarce. A high infiltration of NK cells largely infiltrates kidney cancer tissues [11], which do not effectively eliminate kidney cancer cells, suggesting that NK cells may be heterogeneous and even play a role in promoting tumour immune escape and metastasis. Cancer cells can directly reprogram NK cells to promote metastatic colonisation [28]. Traditionally, NK cells are classified into two subpopulations, CD56dim and CD56bright, based on differences in the density of CD56 expression on NK cells. CD56dim primarily exerts cytotoxic effects, expressing the moderate affinity IL-2 receptor (IL-2R), which has a potent killing activity, whereas CD56bright produces several cytokines and primarily exerts immunomodulatory effects. Evidently, this classification does not explain the high NK cell infiltration in kidney cancer. This is mainly due to the limitations of flow cytometry, which can only detect a limited number of possible markers. scRNA-seq measures thousands of markers in a single cell. Thus, in this study, NK cells were reclassified based on the similarities/differences in their genetic characteristics (Figure 2(a)) into three subpopulations: NK(GZMH), NK(EGR1), and NK(CAPG). Compared to that in the normal kidney cells, the NK(EGR1) and NK(CAPG) subpopulations were abnormally elevated in ccRCC and could mediate ccRCC metastasis, such as Hodgkin's lymphoma, T-cell leukaemia, and Ki-1+ anaplastic large cell lymphoma development in kidney cancer (Figures 4(c) and 4(d)). Thus, the NK cell subpopulations NK(EGR1) and NK(CAPG) are promising as therapeutic targets for metastasis in ccRCC. However, further investigation is needed to elucidate the exact mechanisms involved, and relevant genetically modified animal models are needed to evaluate the status of metastasis in ccRCC.

## 5. Conclusions

The scRNA-seq technique is a powerful tool to discover the heterogeneity of NK cells in cancerous biopsies. In this study, we identified 3 subpopulations of NK cells in surgically isolated ccRCC biopsies, based on their genetic profiles. Furthermore, 2 of them should be taken as therapeutic targets for metastasis. This discovery shed lights in improving immunotherapy against ccRCC.

## Data Availability

The datasets and code generated and analyzed in this study are available from the corresponding author on request.
